# Roles of Nrf2 in Liver Diseases: Molecular, Pharmacological, and Epigenetic Aspects

**DOI:** 10.3390/antiox9100980

**Published:** 2020-10-13

**Authors:** Marina Galicia-Moreno, Silvia Lucano-Landeros, Hugo Christian Monroy-Ramirez, Jorge Silva-Gomez, Jorge Gutierrez-Cuevas, Arturo Santos, Juan Armendariz-Borunda

**Affiliations:** 1 Instituto de Biologia Molecular en Medicina, Centro Universitario de Ciencias de la Salud, Universidad de Guadalajara, Guadalajara 44340, Jalisco, Mexico; marina.galicia@academicos.udg.mx (M.G.-M.); silvia.lucano@gmail.com (S.L.-L.); hugo.monroyram@academicos.udg.mx (H.C.M.-R.); xntonio.silva@gmail.com (J.S.-G.); gutierrezcj05@gmail.com (J.G.-C.); 2 Tecnologico de Monterrey, Escuela de Medicina y Ciencias de la Salud, Zapopan 45201, Jalisco, Mexico; arturo.santos@tec.mx

**Keywords:** antioxidants, liver damage, Nrf2, epigenetics

## Abstract

Liver diseases represent a critical health problem with 2 million deaths worldwide per year, mainly due to cirrhosis and its complications. Oxidative stress plays an important role in the development of liver diseases. In order to maintain an adequate homeostasis, there must be a balance between free radicals and antioxidant mediators. Nuclear factor erythroid 2-related factor (Nrf2) and its negative regulator Kelch-like ECH-associated protein 1 (Keap1) comprise a defense mechanism against oxidative stress damage, and growing evidence considers this signaling pathway as a key pharmacological target for the treatment of liver diseases. In this review, we provide detailed and updated evidence regarding Nrf2 and its involvement in the development of the main liver diseases such as alcoholic liver damage, viral hepatitis, steatosis, steatohepatitis, cholestatic damage, and liver cancer. The molecular and cellular mechanisms of Nrf2 cellular signaling are elaborated, along with key and relevant antioxidant drugs, and mechanisms on how Keap1/Nrf2 modulation can positively affect the therapeutic response are described. Finally, exciting recent findings about epigenetic modifications and their link with regulation of Keap1/Nrf2 signaling are outlined.

## 1. Introduction

Liver diseases represent a critical health problem with 2 million deaths worldwide per year, mainly due to cirrhosis and its complications as a result of damage induced by chronic and excessive alcohol consumption, obesity, hepatitis B virus (HBV), and hepatitis C virus (HCV), which represent the main etiological factors for liver damage development [1,2]. Several cellular and molecular mechanisms are involved in the pathogenesis of liver injury; one of them is oxidative stress, which plays an important role in the initiation and progression of liver disease. This damage mechanism occurs when the balance between reactive oxygen species (ROS) and antioxidants defenses is tilted towards pro-oxidant substances; this in turn leads to damage to lipids, proteins, and DNA [3]. The liver has a comprehensive defense system against oxidative damage; one component is the response exerted by nuclear factor-erythroid 2-related factor 2 (Nrf2) that modulates the expression of phase II detoxifying enzyme genes and antioxidant-response genes by binding to antioxidant response elements (ARE) [3].

Nrf2 is a pivotal player in the preservation of cellular homeostasis. In liver, Nrf2 has an important protective role during inflammation, fibrogenesis, and carcinogenesis [4]. In normal conditions, Nrf2 is present in cytoplasm, interacting with the actin-binding protein Kelch-like ECH-associated protein 1 (Keap1). When oxidants are present, Nrf2 escapes from Keap1 inhibition, and accumulates in the nucleus, thereafter interacting with ARE sequences to act as a transcriptional activator of ARE-responsive genes such as hemeoxygenase-1 (HO-1), glutathione-S-transferase (GST), glutathione peroxidase (GPx), NAD(P)H quinone oxidoreductase 1 (NQO1), superoxide dismutase (SOD), catalase (CAT), and glutathione reductase (GR), to name but a few [5]. Multiple studies have established a connection between Nrf2 and different liver diseases including alcoholic liver disease, metabolic dysfunction-associated fatty liver disease (MAFLD), non-alcoholic steatohepatitis (NASH), viral hepatitis, fibrosis, cirrhosis, and hepatocellular carcinoma (HCC). A thorough understanding of molecular mechanisms modulated by Nrf2 could facilitate new therapeutic strategies for the treatment of liver diseases [3].

On the other hand, several studies have suggested that epigenetic changes, defined as heritable changes to gene expression without affecting DNA sequence, can regulate Keap1/Nrf2 activity [6]; DNA methylation, histone modifications, non-coding RNAs, and chromatin remodeling are implicated in the regulation of this nuclear factor; thus, targeting these epigenetic changes might be useful for modulating the Keap1/Nrf2 signaling pathway and, eventually, for treating liver disease [7].

The aim of this review is to provide an up-to-date overview of the Keap1/Nrf2 signaling pathway and its involvement in the development of main liver diseases such as alcoholic liver damage, viral liver diseases, steatosis, steatohepatitis, cholestatic damage, and liver cancer. In addition, the main current pharmacological therapies and epigenetic modifications related to this transcription factor will be described. 

## 2. ROS, Antioxidant Defenses, and Liver Damage

Development of liver damage is characterized by necrosis of parenchymal cells, exacerbated inflammatory response, and alterations in the composition of the extracellular matrix (ECM) [8]. At the cellular level, hepatic stellate cells (HSCs) and Kupffer cells (KCs) play an important role in hepatic damage, as do also oxidative mediators, cytokines, and chemokines [8].

ROS include molecules derived from oxygen reduction, such as molecules or atoms that have unpaired electrons and are unstable and highly reactive because they can react with adjacent molecules subtracting electrons. Oxidative intermediaries can generally be classified into oxygen-centered radicals, such as superoxide radical, hydroxyl radical, and peroxyl radical, and oxygen-centered non-radicals, such as hydrogen peroxide and singlet oxygen [9]. Production of all these molecules is part of aerobic metabolism in humans, which, in a certain way, is important for some physiological functions such as signal transduction pathways and defense against microorganisms (neutrophils, eosinophils, and macrophages during inflammation), among others [10]. 

In the liver, mitochondria, cytochrome metabolism, microsomes, and peroxisomes are the main endogenous sources of ROS, which, in turn, can produce harmful effects on lipids, proteins, and DNA, altering their functions [8]. Moreover, oxidative damage is able to modulate cell pathways that regulate gene transcription, protein expression, cell apoptosis, HSC activation, and other mechanisms that favor the development of liver diseases [10]. KCs are the cellular type most affected by oxidative damage; however, HSCs and endothelial cells are also affected. In response to damage by oxidative processes, KCs synthesize a variety of cytokines that subsequently favor an increase in inflammation and apoptotic response. On the other hand, the synthesis and accumulation of collagen is stimulated when HSC are activated by ROS [11].

Antioxidants are agents that postpone or prevent the oxidation of oxidizable substrates and scavenge ROS. The human body has different mechanisms to counteract oxidative damage, and these defenses can be both enzymatic and non-enzymatic [9]. Enzymatic defenses, such as CAT, SOD, and GSH-Px, and non-enzymatic antioxidants systems, such as GSH, play an important role in the prevention of liver damage [8].

## 3. Molecular Aspects of Keap1/Nrf2 Signaling

### 3.1. Structural Domains of Keap1 and Nrf2

The liver is the primary organ responsible for the metabolism of xenobiotics, and for this reason, it possesses an extensive and varied set of antioxidant defense mechanisms. The Keap1/Nrf2 pathway is the major system responsible for maintaining liver homeostasis when this organ suffers oxidative damage [9]. Nrf2 regulates the expression of proteins related with phase II metabolic processes such as NADPH, NQO1, GST, GSH-Px, ferritin, HO-1, and other antioxidant genes that prevent liver injury [12]. Nrf2 is a protein constituted by 605 amino acids and seven functional domains called Nrf2-ECH homology (Neh) 1–7, which are responsible for maintaining Nrf2 stability and regulating its transcriptional activity [13,14]. The N-terminal Neh2 domain contains two important motifs, DLG and ETGE, which are critical for the interaction between Nrf2 and its negative regulator, Keap1 [14]. The Neh1 domain contains a basic leucine zipper motif, crucial for the proper binding of Nrf2 to the ARE sequence; this domain also can interact with an E2-ubiquitin conjugating enzyme (UbcM2) to regulate Nrf2 stability [13]. On the other hand, the C-terminal Neh3 domain is a transactivation domain that recruits chromo-ATPase/helicase DNA-binding protein 6 (CHD6) [12], while Neh4 and Neh5 represent other transcription activation domains that recruit cAMP response element-binding protein (CREB)-binding protein (CBP) and receptor-associated coactivator 3 (RAC3) and facilitate Nrf2-mediated transcription [15]. The Neh6 domain represents a binding site for the β-transducin repeat-containing protein (β-TrCP) that modulates Nrf2 degradation independently of Keap1 [13]. Finally, the Neh7 domain interacts with retinoic X receptor α, thus repressing Nrf2. 

Keap1, the main intracellular regulator of Nrf2, is a 624-aminoacid protein that functions as an adapter for Cul3-Rbx E3 ubiquitin ligase complex, and is composed by five domains: a N-terminal domain, a broad complex, tramtrack and bric-a-brac (BTB) domain, an intervening region (IVR), a double-glycine repeats (DGR) domain, and a C-terminal domain, each with an important role in Nrf2 activity inhibition [16,17]. Notably, Keap1 binds to the N-terminal Neh2 domain of Nrf2 via both the DLG and ETGE motifs. In response to oxidative stimuli, the DLG motif in Nrf2 is released from the DGR domain in Keap1, thus blocking Nrf2 ubiquitination and subsequent degradation. The IVR domain conforms to the consensus sequence of a nuclear export signal, which is important for the cytoplasmic localization of Keap1 [18].

### 3.2. Mechanisms of Nrf2 Regulation

In normal conditions, Nrf2 is located in the cytoplasm bound to its inhibitor protein Keap1 and is rapidly degraded by the ubiquitin-proteasome pathway [19]. However, under oxidative conditions, the Keap1-Nrf2 interaction is impaired, leading to Nrf2 stabilization and accumulation in the nucleus, where it heterodimerizes with one of the small musculoaponeurotic fibrosarcoma oncogene homolog (small Maf, sMAF) proteins [13]. These Nrf2-sMaf heterodimers recognize ARE sequences, leading to the transcription of ARE-responsive genes such as HO-1, NQO1, GST, GSH-Px, glutamate-cysteine ligase catalytic subunit (GCLC), and extracellular SOD, all of which are important to counteract oxidative damage [20]. Furthermore, Nrf2-sMaf complexes play important roles in modulating anti-inflammatory responses, autophagy, and proteasome activity [21].

Inactivation of Nrf2 is mediated mainly by Keap1, which facilitated the poly-ubiquitination of Nrf2 by the Cullin 3-Ring box protein (Cul3-Rbx) complex, and consequently Nrf2 is degraded by the 26S proteasome [22]. Human Keap1 contains 27 cysteine residues, of which the major ones involved in stress sensing are Cys151, Cys273, and Cys288 [23]. Under oxidative conditions, ROS can modify Keap1 cysteines via an electrophile reaction, leading to the formation of adducts that prevent Nrf2 ubiquitination and favor its nuclear translocation and the transcriptional induction of Nrf2 target genes [24].

**Figure 1:**
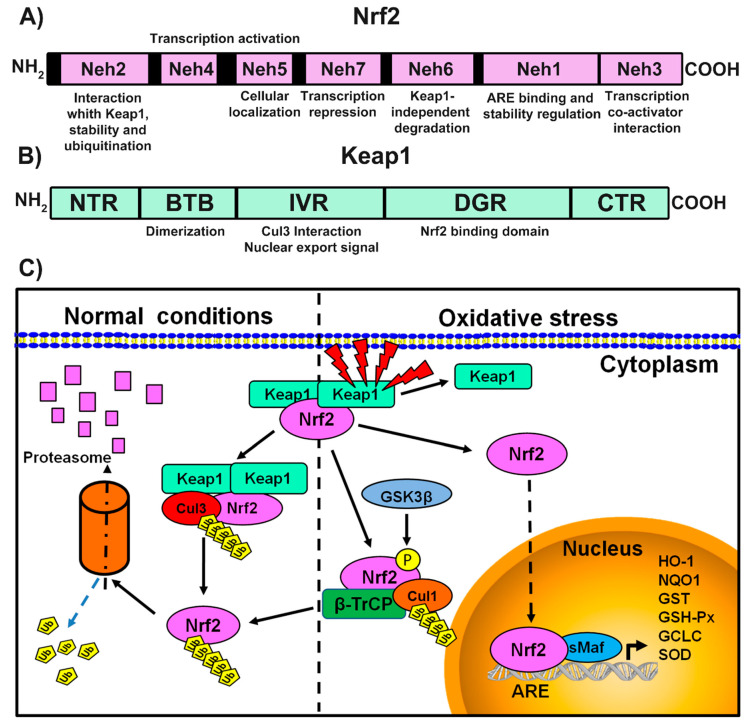
Schematic representation of the primary structure of Nrf2 (**A**) and Keap1 (**B**) domains and their respective functions. (**C**) Representation of regulatory mechanisms in Keap1/Nrf2 signaling. In normal conditions, Nrf2 remains inactive bound to its endogenous inhibitor Keap1; this heterodimer binds the Cul3-Rbx E3 ubiquitin ligase complex that triggers Nrf2 degradation by the proteasome. Under oxidative stress conditions, Nrf2 is released from Keap1 and translocates to the nucleus, forming a heterodimer with sMaf. Nrf2-sMaf heterodimers bind to ARE sequences promoting the expression of antioxidant genes. Alternatively, when Nrf2 is phosphorylated by GSK-3, β-TrCP mediates its interaction with a Cul1 ubiquitin ligase complex (Skp1-Cul1-Rbx1/Roc1) to promote the proteasomal degradation of Nrf2, thus inhibiting the expression of cytoprotective genes.

Alternatively, Nrf2 activity can be controlled by a proteasomal degradation mechanism mediated by the serine/threonine protein kinase glycogen synthase kinase 3 (GSK-3) and the E3 ligase adapter β-TrCP [25]. β-TrCP is a substrate receptor for S-phase kinase-associated protein 1 (Skp1)-Cul1-Rbx1/Regulator of cullins-1 (Roc1) ubiquitin ligase complex that targets Nrf2 for ubiquitination and proteasomal degradation, while GSK-3 is an important protein related with Keap1-independent Nrf2 stabilization and regulation. Active GSK-3 can phosphorylate Nrf2 in its Neh6 domain to facilitate the recognition of Nrf2 by β-TrCP, promoting Nrf2 protein degradation [12]. Lastly, an additional degradation system can regulate Nrf2 activation through the E3 ubiquitin ligase Hrd1, which is part of the inositol-required protein 1 pathway of the unfolded protein response [25]. A schematic illustration of the aforementioned Keap1 and Nrf2 domains and regulatory mechanisms is depicted in Figure 1.

## 4. Nrf2 Connection with Liver Diseases

### 4.1. The Role of Nrf2 in Metabolic Dysfunction-Associated Fatty Liver Disease (MAFLD)

MAFLD includes NASH, fibrosis, cirrhosis, and hepatocellular carcinoma [26,27]. Clinical and experimental data suggest that fatty liver can not only induce serious tissue damage and even cancer, but can also cause cardiovascular diseases [28,29]. Decades ago, a “two-hit hypothesis” was proposed to illustrate the pathogenesis of MAFLD. The first hit is due to insulin resistance, which stimulates liver steatosis with increased hepatic lipogenesis and impaired degradation of free fatty acids (FFAs). Lipid accumulation leads to liver inflammation and cell death by a second pathogenic insult, which generates oxidative stress. Currently, a new theory implicates a “multiple-hit hypothesis” for MAFLD, which culminates in NASH and fibrosis [30]. The excess of palmitic acid increases ROS generation through activation of NADPH oxidase or alteration of the mitochondrial electron transport chain [31], as well as activation of nuclear transcription factor kappa B (NF-κB) [32]. Moreover, peroxisomes produce H_2_O_2_ as a product of fatty acid oxidation [30], and a study using human liver cells showed that H_2_O_2_ decreased the expression of Peroxisome proliferator activated receptor alpha (PPARα) as well as its target genes Carnitine palmitoyltransferase 1A (*CPT-1*) and Acyl-CoA oxidase (*ACOX*) that are involved in fatty acid oxidation. Additionally, H_2_O_2_ upregulates the expression of sterol regulatory element-binding protein-1c (SREBP-1c), with a consequent increase in fatty acid synthase (FAS), thus promoting lipid accumulation [30]. The induction of SREBP-1c, which is a transcriptional activator of lipogenic enzymes such as stearoyl coenzyme-A desaturase1 (SCD1) and fatty acid synthase (FAS), plays a key role in the pathogenesis of MAFLD via an increased rate of lipid synthesis [33]. Endoplasmic reticulum (ER) stress, which is triggered when the folding capability of the ER fails to accommodate the load of unfolded proteins, has been found to contribute to the pathogenesis of MAFLD under obese conditions [34]. ER-stress-induced SREBP activation has been demonstrated under diverse experimental conditions [35]. In addition, hepatic triglyceride accumulation through ER stress-induced SREBP-1 activation is regulated by ceramide synthases [36]. In the complex process of assembly and secretion of very low-density lipoprotein (VLDL), the microsomal triglyceride transfer protein (MTP) facilitates Apolipoprotein B (ApoB) secretion, in part by addition of lipid to the polypeptide as ApoB translocates into the lumen of the ER. Thereby, MTP allows greater incorporation of triglyceride into existing ApoB-containing lipoproteins, thus yielding larger particles [37]. Patients with MAFLD show increased mRNA expression of both MTP and apoB. However, severe insulin resistance is associated with decreased MTP, suggesting that progression of liver disease is accompanied by loss of MTP expression. VLDL secretion persists during pathological liver ER stress and MAFLD, perhaps until hepatic damage impacts the capacity of the secretory pathway to produce VLDL [37]. On the other hand, ER stress induces hepatic steatosis via increased expression of liver VLDL receptor through direct binding of Activating transcription factor-4 (ATF4) to the promoter region of the gene encoding the VLDL receptor (VLDL-R), ultimately leading to deposition of triglycerides [38]. Because oxidative stress is important in the progression of MAFLD [9], it is a plausible therapeutic target to prevent MAFLD progression.

In subjects with MAFLD, Nrf2 activation occurs in response to disease development, but the expression of antioxidant enzymes seems to decrease as MAFLD progresses [39]. Nrf2-knockout (Nrf2-KO) cells have lower levels of glutathione, whereas Keap1 deficiency promotes glutathione upregulation [24,40]. The induction of the Keap1/Nrf2 signaling pathway may provide protection to hepatic cells from oxidative stress and avoid the progression of MAFLD [41]. A microarray study revealed that both genetic and pharmacologic activation of Nrf2 resulted in induction of pathways beyond detoxification and cytoprotection, including genes of lipid metabolism [9,40]. Nrf2 activation also inhibits the transcription of IL-6 and IL-1β [40] and reduces hepatic glucose production in humans [32]. Osteocalcin, scutellarin, apigenin, and berberine were found to improve MAFLD by activating the Keap1/Nrf2 antioxidant system [4,42].

The p62 protein or sequestosome 1 (SQSTM1) protects against lipotoxicity through activation of the non-canonical Keap1/Nrf2 pathway in hepatocytes; in the absence of p62, cells are most susceptible to oxidative stress [43]. Autophagy is initiated by Unc-51 Like Autophagy Activating Kinase 1 (ULK1) complex, and it was showed that SQSTM1 induces ULK1 phosphorylation, which leads to induction of macroautophagy, and thereby to Keap1 degradation and Nrf2 activation [44]. 

Nrf2 activation ameliorates methionine- and choline-deficient (MCD) diet-induced hepatic steatosis, inhibiting Cluster of differentiation 36 (CD36), Fibroblast growth factor 21 (Fgf21), and PPARα expression in the liver of mice [45], whereas Nrf2 deletion increases PPARα expression [3,40,46]. Mouse embryonic fibroblasts extracted from Keap1-knockdown (Keap1-KD) mice exhibit impaired adipogenesis, which identifies Nrf2 as a negative regulator of lipid metabolism [47]. The induction of Nrf2 via Keap1-KD or sulforaphane treatment decreased the expression levels of PPAR-gamma (PPARγ), CCAAT/enhancer-binding protein alpha (C/EBPα) and fatty acid-binding protein 4 (FABP4) in mouse embryonic fibroblasts [48]. Keap1-KD mice have decreased lipid deposition, and Nrf2 activation by Keap1-KD increased AMP-activated protein kinase (AMPK) phosphorylation in hepatocytes [49]. Conversely, the AMPK pathway is downregulated in Nrf2-KO mice, including acetyl coenzyme A carboxylase (ACC) phosphorylation [39]; these mice were unable to adapt to hepatic oxidative stress, which accelerated MAFLD development [46]. These results are consistent with the observed elevation in lipid deposition in the liver of Nrf2-KO mice [3]. Nrf2-KO mice also show increased expression of lipogenic genes in response to high-fat diet (HFD) [41], findings corroborated by an independent proteomic analysis [50]. 

### 4.2. Controversies in the Role of Nrf2 in Energy Metabolism

The expression of Nrf2 was elevated in livers of mice fed an HFD for 12 weeks [41]. In contrast, C57BL/6J mice with hepatic steatosis showed decreased mRNA expression of AMPK-PGC-1α signaling components, as well as Nrf2 and β-ATP synthase [51]. In rats fed an HFD, *Lactobacillus mali* APS1 reduced the levels of hepatic lipids by regulating the expression of sirtuin-1 (SIRT-1)/peroxisome proliferator-activated receptor gamma coactivator-1 alpha (PGC-1α)/SREBP-1, and it also increased the hepatic antioxidant activity via induction of Nrf2 and HO-1 [51]. Nrf2-KO mice fed an HFD showed a downregulation of lipid metabolic genes, preventing hepatic steatosis [52]. Similarly, in another study, Nrf2-KO mice fed a chronic HFD exhibited decreased hepatic lipid accumulation and decreased hepatic steatosis [40]. In contrast, Nrf2-KO mice fed an HFD for 4 weeks exhibited increased expression of lipogenic genes and hepatic FFAs content [41]. Nrf2 negatively regulates FABP1/4/5, which participate in the uptake of FFAs and their transport from the blood into hepatocytes [45,46,53]. CD36 was identified as a downstream target of Nrf2 [52], and CD36 was found to be upregulated in hepatocyte-specific Keap1-KO mice, while the transcript levels of FABP1 were significantly reduced [53]. The expression levels of CPT1/2 were increased in wild-type mice fed an HFD with iron; conversely, they were diminished in Nrf2-KO mice [3]. However, Nrf2-KO mice fed an HFD showed increased CPT1 levels compared to wild-type mice [46], which can contribute to increased steatosis. Besides, Keap1-KD mice fed a long-term (24 weeks) HFD showed hepatic inflammation and steatosis, without affecting the protein level of Glut4 [47,54]. However, 8-week-old Keap1-KD *ob*/*ob* mice showed a reduction in Glut4 protein levels and in Akt phosphorylation [48]. In contrast, mice deficient in Nrf2 fed a high-fat Western diet for 12 week showed improved glucose tolerance [55]. Mice with cell-specific deletion of Nrf2 in adipocytes or hepatocytes and fed an HFD for 6 months showed increased body fat content. Mice with deletion of Nrf2 in adipocytes showed a partially deteriorated glucose tolerance, higher fasting glucose levels, and higher levels of cholesterol and non-esterified fatty acids. Meanwhile, mice with deletion of Nrf2 in hepatocytes demonstrated lower insulin levels and tended toward improved insulin sensitivity without affecting liver triglyceride accumulation [56]. Interestingly, Keap1-KD mice fed an HFD for 3 months showed partial protection from obesity [57].

To date, there is no clear explanation for the aforementioned discrepancies in the role of Nrf2 during diet-induced obesity. The observed apparent contradictions could be due to differences in experimental design, including the gender, genetic background, and age of the mice, as well as the mechanism(s) involved in Nrf2 activation in the various settings.

### 4.3. Nrf2 as a Potential Therapeutic Target for Non-Alcoholic Steatohepatitis

Oxidative stress is the main component of hepatocellular injury and may worsen inflammation and fibrosis in the liver of patients with NASH [58]. Additionally, elevated activity of CYP2E1 is an important generator of free radicals in NASH [59]. The profile of NASH includes a decrease of liver superoxide dismutase and catalase, with increased lipid peroxidation within hepatocytes. Regarding lipid peroxidation, nucleotide and protein synthesis are impaired, thus inducing apoptosis, inflammation, and liver fibrosis [60]. The unfolded protein responses (UPR) plays an important role in cellular stress and inflammation in NASH [51]. 

Nrf2 plays a role key in NASH, and its activation has been found to protect against NASH [4,9,60]. Nrf2-KO mice are susceptible to develop NASH when fed an MCD diet or an HFD [3]. Nrf2 activation inhibits liver X receptor-alpha (LXRα) activity and LXRα-dependent liver steatosis [41]. Genetic activation of Nrf2 in Keap1-KD mice has been reported to inhibit steatohepatitis [9]. However, Keap1-KD mice fed an HFD for 24 weeks showed hepatic steatosis and inflammation [54]. In hepatocyte-specific Nrf2-overexpressing mice, upregulation of genes such as Gpx2, thioredoxin 1 (Trx1) and NQO1 was observed independently of normal chow diet or an MCD diet [53]. This finding is in line with a previous study showing that hepatic Nrf2 overexpression in mice protects against oxidative stress induced by long-term exposure to an MCD diet [61]. Data obtained from patients with NASH reported that the nuclear abundance of Nrf2 protein was increased in the liver, and the hepatic expression of γ-glutamylcysteine synthetase, Gpx2, Txn1, and HO-1 was upregulated [62]. 

Nrf2 activation exerts potent anti-inflammatory effects [63], and Nrf2-KO mice fed an HFD developed hepatic insulin resistance through an increase in the levels of NF-κB, IL-6, and TNF-α [64]. Nrf2-KO mice fed an MCD diet for 14 days also showed an increase of NF-κB [3]. Similar results were found in the livers of Nrf2-KO mice, with a constitutive activation of NF-κB and c-Jun N-terminal kinase (JNK) [46]. However, no differences were noted between hepatocyte-specific Keap1-KO mice and wild-type mice in inflammatory F4/80- and CD11b-positive cells or pro-fibrogenic genes [53]. In addition, transgenic mice expressing Nrf2 in hepatocytes and fed an MCD diet during 28 days showed increased expression of genes involved in triglyceride export, such as Microsomal Triglyceride Transfer Protein (MTTP), and β-oxidation, such as CPT2, but no differences in oxidative stress and inflammation, which were both increased similar to control mice [61], suggesting that hepatocytes alone are incapable of inducing inflammation. Interestingly, hepatocytes from mice with double liver-specific KO of *c-met* and *Keap1* (in which Nrf2 is overactivated) and fed an MCD diet for 4 weeks, displayed increased liver mass but decreased triglyceride deposition [65].

Green tea extract and ezetimibe have been found to promote the protective effect of Nrf2 against lipid deposition and inflammation in NASH through p62-dependent activation of Nrf2 [4]. The herbal supplement protandim has been shown to activate Nrf2 in human trials, and to increase superoxide dismutase and catalase activity in erythrocytes [60]. The acetylenic tricyclic bis(cyano enone), TBE-31, reversed insulin resistance and decreased liver steatosis, fibrosis, and oxidative stress in the livers of C57BL/6 mice; these effects were not observed in Nrf2-KO mice, demonstrating that they were Nrf2-dependent [40]. 

### 4.4. Nrf2 and Alcoholic Steatohepatitis

Alcoholic liver disease includes simple fatty liver (steatosis) and more severe forms of liver damage, including alcoholic steatohepatitis, cirrhosis, and HCC [41]. Oxidative stress and lipid accumulation play important roles in alcoholic liver injury [41]. Chronic ethanol consumption results in depletion of total and mitochondrial reduced GSH, which contributes to ROS accumulation, increased lipid peroxidation, and induction of cell necrosis and/or apoptosis [41]. These effects are more pronounced in Nrf2-KO mice, in ethanol treatment caused marked steatosis and inflammatory response mediated by KCs [41,60]. In Keap1-KD mice, Nrf2 activation prevented alcohol-induced oxidative stress and deposition of FFAs in the liver [9]. Nrf2 activation was also protective against alcohol-induced liver fibrosis and hepatotoxicity, whereas Nrf2-KD mice showed alcohol-induced hepatocyte necroptosis [4]. Moreover, Nrf2 activation by 3H-1,2 dithiole-3-thione (D3T) reduced generation of ethanol-induced ROS and apoptosis [4], and the protective effect of Nrf2 was observed in both in vivo and in vitro models; for instance, sulforaphane administration improved alcohol-induced liver steatosis [9]. Solanesol increased the expression levels of HO-1 and heat shock protein 70 (Hsp70), mediated by Nrf2 and heat shock factor 1 (HSF1), respectively, and protected human hepatic L02 cells from ethanol-induced oxidative damage [66]. Moreover, a study in zebrafish larvae found that *Lactobacillus plantarum* activates the Keap1/Nrf2 pathway, and promotes the activities of SOD, CAT, HO-1, and GSTK1, which reduced oxidative stress in the liver induced by alcohol [67]. Finally, inhibition of autophagy reversed the alcohol-induced activation of HSCs through stimulation of Keap1/Nrf2 signaling [68].

Studies in Nrf2-KO mice demonstrated most convincingly the protective role of Nrf2 against ethanol-induced damage. In mice treated with ethanol, loss of Nrf2 was shown to result in severe steatosis, liver inflammation, and mortality [3]. Lastly, Nrf2 activation induced by oxidative stress is considered to positively modulate the expression of VLDL-R, which contributes to alcoholic liver disease [4]. In summary, Nrf2 has a protective role against ethanol-induced oxidative stress and could thus be a promising target for the treatment of alcoholic liver disease.

### 4.5. The Role of Nrf2 in Hepatic Fibrosis

Hepatic fibrosis is a common reversible wound-healing response to chronic liver injury and inflammation and is mainly attributed to HSC activation that occurs after long-term liver damage by alcohol, HBV, HCV, and non-alcoholic fatty liver. When aggravated, hepatic fibrosis can progress to cirrhosis and hepatocarcinoma [69]. 

Overexpression of Nrf2 in hepatocytes of c-met/Keap1 KO mice, resulted in less oxidative stress, inflammatory cells, and fibrosis moderately reduced [65]. In contrast, Nrf2 deficiency worsened carbon tetrachloride (CCl_4_)-induced liver inflammation and fibrosis [3,41]. Ligutrazine is a compound known for its antifibrotic effects, and in Nrf2-KD mice, the effects of ligustrazine on hepatic fibrosis were decreased [70].

Increased Nrf2 expression in response to ginsenoside Rg1 and chebulic acid resulted in inhibitory effects on HSC activation and experimental hepatic fibrosis [9,71]. Pharmacologic activation of Nrf2 by TBE-31 decreased liver fibrosis in high-fat plus fructose-fed mice with NASH [72]. Other Nrf2 activators such as the thiol-reactive agent oltipraz (OPZ) and NK-252 (1-(5-(furan-2-yl)-1,3,4-oxadiazol-2-yl)-3-(pyridin-2-ylmethyl)urea) significantly attenuated the progression of hepatic fibrosis in a rat model of NASH [3]. In liver, the antifibrotic effect of Nrf2 is due to the promotion of fibroblast differentiation [9], and an inhibitory effect on TGF-β1 has been shown in an HSC cell line [4]. Conversely, Nrf2-KD induces HSC activation with an increase of α-SMA and induction of the TGF-β1/Smad pathway [73]. However, it was reported that Nrf2 activation by miRNA-200a inhibited HSCs stimulation in a TGF-β1-independent manner [74]. Pro-fibrotic compounds such as TGF-β1, methotrexate and thioacetamide induce both HSC activation and Nrf2 activation in a human 3D-multicellular model of liver fibrosis [75]. Nrf2-KO mice under long-term CCl_4_ treatment showed prolonged inflammatory and profibrogenic responses. In a different model, sulforaphane suppressed hepatic fibrosis induced by bile duct ligation (BDL) in mice [41]. In summary, there is evidence that activation of the Keap1/Nrf2 signaling pathway may be an effective strategy for the prevention of liver fibrosis.

### 4.6. Effects of Nrf2 on Hepatic Cirrhosis

Oxidative stress, UPR, and lipid peroxidation are associated with the pathogenesis of liver cirrhosis [51]. The mRNA expression levels of Nrf2 were reported to be increased in cirrhosis compared to normal liver [69]. However, during end-stage liver cirrhosis in mice, hepatic Nrf2 is inhibited as a result of activation of the Ire1a-Xbp1 arm of the UPR, which promotes disease [24,40]. These findings indicate that activation of Nrf2 confers protection against hepatic cirrhosis [24,41]. Additionally, treatment with ursodeoxycholic acid (UDCA) enhanced hepatic expression of Nrf2 and increased the protein abundance of the Nrf2 targets Trx and Trxr-1 in patients with primary biliary cirrhosis [60]. Therefore, activation of Nrf2 has potential therapeutic utility in the management of liver cirrhosis.

### 4.7. Role of Nrf2 in Hepatic Cholestasis

Cholestasis is characterized by impaired hepatic bile flow, which leads to accumulation of bile acids and other chemicals in liver and blood. BDL, a model of extrahepatic obstructive cholestasis, causes induction of inflammatory liver injury and fibrosis [76]. In addition, BDL induces several antioxidant genes regulated by Nrf2, which likely defend against the oxidative stress generated in the liver during this procedure [77]. 

Nrf2-KO mice with BDL showed reduced GSH excretion, and higher levels of intrahepatic bile acids, Mrp3 and Mrp4, whereas bile acid synthetic enzymes CYP7a1 and CYP8b1 were decreased [60]. In contrast, BDL-induced liver injury wss diminished in Keap1-KD mice thanks to enhancement of antioxidative stress systems, along with Mrp efflux transport [40]. Exposure to lithocholic acid (LCA) at high levels causes cholestatic liver injury in rodents. Nrf2-KO mice treated with LCA show severe multifocal liver necrosis [60]. On the other hand, Nrf2 activators induced Mrp efflux transporters in rodent liver, and Nrf2 is well-known to regulate the induction of hepatic detoxification and antioxidant mechanisms [9,24,41]. In wild-type mice, UDCA improves a variety of cholestatic liver diseases through increases in nuclear levels of Nrf2 and induction of Mrp2, Mrp3, and Mrp4 [60]. As mentioned above, UDCA treatment in patients with primary biliary cirrhosis enhanced hepatic expression of Nrf2 and increased the protein abundance of the Nrf2 targets Trx and Trxr-1 [24,41]. The compound α-naphthylisothiocyanate (ANIT) is known to induce intrahepatic cholestasis; its hepatotoxicity was similar between wild-type and Nrf2-KO mice; however, Nrf2-KO mice showed less accumulation of bile acids in serum compared to wild-type mice. Aditionally, Bsep, Mdr2, and Mrp3 efflux transporters were increased by ANIT in wild type mice but not in Nrf2-KO mice [78]. Ethyl acetate extract may ameliorate the cholestasis and liver injury caused by ANIT in rats by inducing farnesoid X receptor (FXR) and suppressing the Keap1/Nrf2 and NF-κB signaling pathways [79]. It is possible that these conflicting results could be dependent on the specificities of the cellular context and the animal model used. In summary, the results suggest that Nrf2 activation may be useful for the prevention of cholestatic liver injury.

### 4.8. Role of Nrf2 in Hepatocellular Carcinoma

HCC, one of the most common tumor types in the world, accounts for more than 80% of all hepatic malignancies [4]. The incidence of HCC is higher in patients with HCV-related chronic liver disease compared to patients with NASH [80]. Both male and female Nrf2-KO mice treated with 2-amino-3-methylimidazo[4,5-f]quinoline showed high incidence of liver tumors [60]. In addition, indazolo[3,2-b]quinazolinones damage HCC cells by inhibiting Nrf2 signaling [4]. In a rat model, pomegranate reduced hepatocarcinogenesis via Nrf2 upregulation [60]. HCC shows molecular alterations in the early stage of carcinogenesis, including activation of the Nrf2 pathway that contributes to the progression of preneoplastic lesions along the path of malignant evolution [4]. Furthermore, high expression of Nrf2 has been documented in HCC patient samples [81]. In general, liver cancer often begins in a setting of chronic hepatic inflammation [82]. Nrf2 disruption may contribute to the progression of inflammation and, ultimately, the development and progression of cancer [83]. Loss-of-function mutations in *KEAP1* were identified in multiple cohorts of HCC [82]. In HCC, mutations in *NFE2L2*, the gene that encodes Nrf2, are more common than in Keap1; these mutations occur as late events in HCC, as they were found in advanced stages of human liver carcinogenesis [84].

Mallory–Denk bodies (MDBs) and intracellular hyaline bodies (IHBs) have been described as cytoplasmic inclusions in a subtype of HCC. MDBs are composed of the intermediate filament proteins keratin 8 (K8) and K18, as well as p62 and ubiquitin, whereas IHBs consist of p62 and/or ubiquitin. The presence of IHBs was found to be associated with significantly shorter overall survival in patients with HCC [85]. Several studies have shown that autophagy suppresses liver tumorigenesis; for instance, in early stages of HCC, autophagy was reported to suppress tumor formation by inhibiting inflammation, maintaining genomic stability and repressing p62 accumulation [81]. In HCC, an association between dysfunctional autophagy and Nrf2 activation has been described. Specifically, persistent activation of Nrf2 was associated with accumulation of p62, thereby contributing to HCC progression and inducing robust production of GSH that results in chemoresistance and increases the proliferative capacity of hepatoma cells [86]. Nrf2 overexpression leads to increase expression of the anti-apoptotic factor Bcl-xL, which decreases the expression of the pro-apoptotic factor Bax and the activity of caspase 3/7; thus, Nrf2 contributes to cancer cell survival [86]. In HCC cells, mutations in *NFE2L2* or *KEAP1* activate the Keap1/Nrf2 pathway, increasing the nuclear abundance of Nrf2 and promoting the subsequent activation of its target genes, which results in tumor cell survival and promotion of tumorigenesis [86]. Evidence indicates that Nrf2 also regulates the proliferation, migration, and invasiveness of HCC cells [81]. Nrf2 induces proliferation and invasion of HCC through expression of matrix metalloproteinase-9 (MMP-9) and BCL-xL [81]. Nrf2 overexpression induces the activation of metabolic enzymes such as glucose-6-phosphate dehydrogenase (G6PD) and 6-phosphogluconate dehydrogenase (PGD) that further promote glutamine and glucose metabolism, thereby increasing purine synthesis and ultimately inducing cell proliferation [81]. Finally, Nrf2 activation was associated with HCC progression and metastasis [4]. Several studies showed that miR-340, miR-144, camptothecin, and valproic acid suppress Nrf2 signaling, thereby sensitizing HCC cells to anticancer treatments [4]. Moreover, inhibition of the p62/Keap1/Nrf2 pathway increased the erastin- and sorafenib-induced suppression of HCC [87]. In addition, a genome-wide CRISPR/Cas9-based screening on sorafenib-treated HCC cells was performed to identify genes associated with acquired sorafenib resistance and/or sensitivity in HCC cells. HCC cells with disrupted *KEAP1* were less sensitive than wild-type cells to short- and long-term treatment with sorafenib. *KEAP1* inactivation led to sorafenib, lenvatinib, and regorafenib resistance in HCC cells through induction of Nrf2 target genes and reduction of ROS levels [88]. Other compounds such as the flavonoids apigenin and luteolin, as well as the potent Nrf2 inhibitor chrysin, among others, have been shown to reduce the expression of Nrf2 and its target genes, which suggests that these compounds may be considered as potential anti-cancer agents [86]. Therefore, Nrf2 is a potential molecular target for liver cancer prevention and treatment.

### 4.9. Nrf2 and HCV

HCV may cause chronic hepatitis, steatosis, fibrosis, cirrhosis, and HCC. Several studies correlated cellular stress with HCV infection, and persistence of oxidative stress during HCV replication has been widely observed [89]. The HCV-related proteins, as well as non-structural and structural proteins, were found to trigger production of ROS [3,9,25,41].

There are conflicting results related to interference of HCV with the Keap1/Nrf2 pathway. In HCV-infected cells, Nrf2 activation is mediated through mitogen-activated protein kinases, casein kinase 2, phosphoinositide-3 kinase, and protein kinase C, thus contributing to cell survival against HCV infection [90]. In contrast, an inhibitory effect on the activation of Nrf2 and induction of ARE-dependent genes was reported, related to an increase of sMaf proteins [90,91]. In addition, the transcriptome analysis of HCV-replicating cells showed reduced expression of a variety of Nrf2-dependent genes [90]. In a cell line with Nrf2-KD, HCV infection and steatosis were reduced; further, brusatol, an inhibitor of Nrf2, was found to have anti-HCV effects in vitro [4,89]. Additionally, impaired Nrf2 signaling upon HCV infection was shown to promote elevated ROS levels [89]. Caffeic acid induces Nrf2 and HO-1 and inhibits HCV replication through induction of the IFNα antiviral response and p62-mediated Keap1/Nrf2 signaling [92]. Other compounds such as lucidone, andrographolide, aulforaphane, and celastrol were also shown to inhibit HCV replication through upregulating HO-1 via the Nrf2 pathway [91]. The controversial results on the Nrf2 activation status in HVC infection might be due to differences in experimental designs and conditions.

### 4.10. Nrf2 and HBV

Chronic infection with HBV causes liver inflammation and can promote fibrosis, which can ultimately lead to cirrhosis and HCC [25,89]. Proteins of HBV (HBx) were reported to induce the formation of ROS, and both regulatory proteins of HBV (HBx and LHBs) activate NF-κB, which leads to induction of proinflammatory cytokines [25]. HBV induces a potent activation of ARE-regulated genes via the c-Raf-MEK-Erk signal transduction pathway, which protects infected cells against oxidative damage, maintaining the integrity of the human and viral genomes [93]. However, infection by HBV genotype G suppresses the Nrf2 pathway due to intracellular accumulation of subviral HBsAg particles, and the expression levels of Nrf2 target genes are decreased in HBV/G replicating cells [91]. In summary, regulatory proteins of HBV genotypes have different effects on the Keap1/Nrf2 signaling pathway and the respective virus-associated pathogenetic processes.

## 5. Antioxidant Drugs for Liver Diseases, and the Keap1/Nrf2 Signaling Pathway

There are different compounds with antioxidant capacities that can protect cells from intrinsic and extrinsic cellular stress and that have also shown efficacy in the treatment of liver diseases. These compounds typically act with a great affinity for ROS, donating electrons to counteract their reactivity and maintaining the redox balance [94]. Many plant-derived drugs, such as curcumin, resveratrol, and quercetin, or synthetic compounds, such as pirfenidone and oltipraz, could be good candidates for the treatment of different diseases where oxidative stress is involved [94]. Importantly, several studies have shown the ability of these drugs to prevent liver damage through positive modulation of Nrf2 signaling [24].

### 5.1. Resveratrol (RSV)

The polyphenol RSV is a phytochemical present in fresh grape skin, red wine, peanuts, and berries [95]. Different lines of evidence have highlighted the anti-inflammatory, anti-carcinogenic, and anti-fibrotic properties of RSV, generated through its ability to modulate different cell signaling pathways such as NF-κB, caspases, matrix metalloproteinases, Wnt, SIRT1, PPARγ, insuline-like growth factor-binding protein 3, and cyclooxygenase 2, to name but a few [95,96]. As an antioxidant molecule, RSV promotes the nuclear translocation of Nrf2 [97]. RSV efficacy was observed in an experimental model of HCC, where it prevented lipid peroxidation and accumulation of carbonylated protein, abrogated iNOS induction during hepatocarcinogenesis, and increased Nrf2 expression levels. All these mechanisms make RSV an attractive compound for the prevention of oxidative damage generated during HCC pathogenesis [98]. RSV diminished Nrf2 methylation induced by an HFD in a murine model, thereby diminishing the expression of genes related with hepatic lipogenesis such as FAS and SREBP-1c [99]. Although its efficacy and safety have been demonstrated, in both preclinical and clinical studies, the therapeutic use of RSV has been limited due to its rapid metabolism and poor bioavailability [96].

### 5.2. Curcumin

Curcumin or diferuloylmethane is a natural polyphenol obtained from rhizomes of *Curcuma longa*, which has several pharmacological properties as an antioxidant, anti-inflammatory, anti-fibrogenic, anti-microbial, and anti-carcinogenic compound [100]. As an antioxidant, curcumin is an ideal ROS scavenger; it is also effective in increasing GSH levels and HO-1 expression through Nrf2 modulation [101]. Curcumin was able to activate Nrf2 expression in quinocetone- and furazolidone-induced liver damage, as well as in acute liver damage induced by CCl_4_ [102,103]; it was also able to increase the activity of SOD, CAT, GPx, and GST [104]. Curcumin administration is also effective to reduce lipid deposition through induced expression of FXR and Nrf2 in an experimental model of alcoholic liver damage [105]. Novel discoveries about the effects of curcumin concern its ability to promote DNA demethylation and inhibit histone deacetylases, thereby suppressing HCC development [106]. In clinical studies, the efficacy of curcumin has been shown in patients with cancer or diabetes; however, its bioavailability is low [107]. In a clinical trial of NAFLD, curcumin administration (1000 mg/day for 8 weeks) was safe and able to decrease the fat liver content [108]. Curcumin administration is thus effective for the treatment of diseases related with oxidative processes through cellular signaling pathways including ERK-p38-MAPK, hepatic Keap1/Nrf2 signaling, AMPK signaling, and lipid metabolism [109].

### 5.3. Quercetin

Quercetin is a flavonoid present in high concentration in apples and onions; it possesses various pharmacological properties, acting as an antioxidant, anti-inflammatory, bacteriostatic, cardioprotective, and anti-carcinogenic compound [110]. Besides its properties as a ROS scavenger, its antioxidant effect in liver damage is exerted trough increasing SOD and CAT gene expression and upregulating HO-1. Additionally, quercetin has the capacity to modulate Nrf2 and, consequently, promote the translocation of Nrf2 to the nucleus and its binding to ARE to increase GSH levels and GPx expression [111]. The main metabolites of quercetin, 3′-*O*-methyl quercetin (3′MQ) and quercetin3-*O*-glucuronide (Q3GA), have shown cytoprotective effects against alcoholic liver damage [110].

### 5.4. Pirfenidone (PFD)

PFD or 5-methyl-1-phenil-2-(1*H*)-pyridone, is an antifibrotic, anti-inflammatory and antioxidant molecule used for the treatment of idiopathic pulmonary fibrosis [112], and its effectiveness has also been evaluated in other fibrotic diseases [113,114]. Studies suggest that PFD is effective to prevent damage response through inhibition of NF-κB signaling, decreasing the pro-inflammatory response, and preventing oxidative damage [115]. As an antioxidant compound, PFD is able to block the reactivity of ROS, and to boost the antioxidant cellular defenses [116,117]. Diverse studies have shown the ability of PFD to modulate Nrf2 and counteract oxidative damage; for example, PFD was found to increase Nrf2, HO-1, and Gpx mRNA and protein expression in bleomycin-induced idiopathic pulmonary fibrosis [118]. In an in vitro model using HepG2 cells and primary HSC cultures, PFD treatment induced the expression of Nrf2 and of antioxidant genes related with GSH synthesis such as GCLM, GCLC, and HO-1 [119].

### 5.5. Other Drugs Related with Nrf2 Signaling that Have Potential Effects in the Treatment of Liver Damage

Oltipraz or 4-methyl-5(pyrazinyl-2)-1-2-dithiole-3-thione is a synthetic dithiolethione that activates Nrf2 and promotes the transcription of several antioxidant genes. This drug promotes GSH biosynthesis and the expression of enzymes related with phase II biotransformation such as NQO1 [120]. In in vivo studies, oltipraz has been shown to prevent liver damage induced by CCl_4_ and acetaminophen [121]. The bile acid UDCA is used for treating several liver diseases such as hepatitis and biliary cirrhosis [122]; it can act as an antioxidant molecule, increasing the gene expression of glutamine-cysteine ligase, a key enzyme in GSH synthesis, and also enhancing Nrf2 expression and promoting its nuclear translocation in a model of cholestatic liver damage [123,124]. Finally, N-acetyl cysteine (NAC) is the acetylated precursor of both the amino acid L-cysteine and GSH and is the main antidote currently used clinically for acetaminophen overdose [125]. Studies have demonstrated its antioxidant capacity and its ability to prevent liver damage [126]. Other antioxidant mechanisms exerted by NAC include its ability to modulate Nrf2 signaling, whereby it can increase Nrf2 and HO-1 mRNA levels to prevent hepatic injury [127].

While all compounds reviewed in this section have shown efficacy and safety in experimental studies and a few of them have also been evaluated in clinical trials with good results, clinical use is often hampered by suboptimal pharmacokinetic properties, as discussed.

## 6. Epigenetic Modifications Regulating Keap1/Nrf2 Signaling

Epigenetics emphasizes mechanisms that regulate gene expression without modifying the DNA sequence. DNA hypermethylation or hypomethylation and histone modification have important roles in gene expression and activity of Keap1/Nrf2 signaling [40].

Several authors have postulated that epigenetic regulation of Nrf2 is a possible therapeutic target in the treatment of diseases related to oxidative stress or drug metabolism. For example, Yu et al. [128] used murine models of prostate cancer and demonstrated that differential DNA methylation regulates Nrf2 expression. In particular, hypermethylation of 5 CpG sites in the *Nrf2* promoter inhibits mRNA expression; however, when cells are incubated with the demethylating agents 5-Aza and TSA, the activity of DNA methyltransferase 1 (DNMT1) and histone deacetylase (HDAC) is inhibited, restoring the mRNA expression levels of Nrf2. Similarly, in humans, methylation of 3 CpG sites has been reported to inhibit Nrf2 expression in prostate cells [129]. The positive effects of mixture of tocopherols, curcumin analogs (FN1), and resveratrol were evaluated in an in vivo model of NAFLD, as well as an in vitro model using different cancer cells lines. These treatments showed a suppressive effect on methylation through DNMT1 and HDAC repression, thereby increasing the expression of Nrf2 and its cytoprotective target genes [99,130,131]. Moreover, hypermethylation of the *Keap1* gene promoter causes Nrf2 overactivation and Nrf2 nuclear accumulation; such responses have been observed in breast, kidney, colon, lung, and head and neck cancer tissues [132].

Histone modifications also have an impact on the Keap1/Nrf2 pathway. Deacetylation of H3 and H4, mediated by HDAC and HDAC2, decreases Nrf2 signaling and the antioxidant response and increases the sensitivity to oxidative stress [6]. Similar effects were shown by trimethylation of histone H3 at lysine 27 (H3K27me3) through action by N-methyltransferase enhancer of zeste homolog 2 (EZH2), which decreased the expression of both Nrf2 and Keap1 [6]. On the other hand, inhibitory treatment directed at HDAC decreases Keap1 expression and increases nuclear translocation of Nrf2 [7]. Finally, SET domain-containing 7 histone-lysine N-methyltransferase (SetD7)-mediated methylation of histone H3 on lysine 4 (H3K4me1) favors the binding of transcription factors such as Sp1 to the *Keap1* promoter, which upregulates its transcription and suppresses the Nrf2 response [6,7].

**Figure 2:**
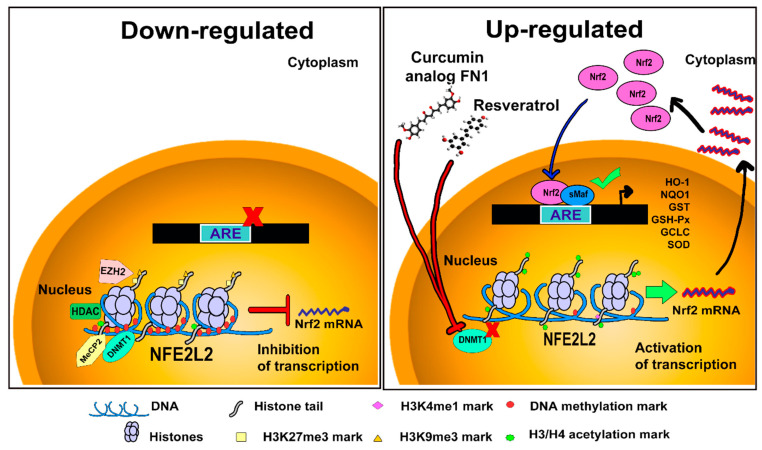
Epigenetic modifications of *NFE2L2*. The left panel shows epigenetic modifications that lead to downregulation of *NFE2L2*. Hypermethylation of both, DNA and histones, reduces the mRNA expression levels of Nrf2. The right panel shows that hypomethylation and acetylation of histones increase the mRNA expression levels of Nrf2 and consequently the activation of cytoprotective genes. Resveratrol and curcumin analog (FN1) reduce the activity of DNMT1 and increase the activity of HDACs, promoting the expression of Nrf2.

The aforementioned epigenetic mechanisms, as well as the antioxidant molecules involved in modulating the activity of the Keap1/Nrf2 signaling pathway, are summarized in Figure 2.

## 7. Conclusions

Regardless of its etiology, the spectrum of chronic liver disease involves different damage processes including oxidative stress, which can be a trigger for inflammation and fibrosis; these can ultimately lead to more severe stages of liver damage, such as cirrhosis or cancer. In recent years, interest has arisen in studying the transcription factor Nrf2 as a potential therapeutic target for the treatment of liver diseases, including epigenetic modifications that favor the activity of this endogenous antioxidant system. At present, it is known that different antioxidant molecules such as pirfenidone, curcumin, quercetin, etc., exert their effects by modulating the Nrf2 pathway; however, few studies have elucidated in detail the molecular modifications exerted by these drugs specifically in the liver.

Due to the complex pathophysiology of liver diseases, there is currently no effective treatment to counteract this damage. It is hoped that studying the antioxidant properties of various compounds can make them good candidates for the treatment of these diseases, especially by focusing beyond their roles as ROS scavenger, and specifically on their effects as modulators of Nrf2 signaling and/or as modulators of epigenetic mechanisms.

## Abbreviations

ACC	Acetyl coenzyme A carboxylase
ACOX	Acyl-CoA oxidase
AMPK	AMP-activated protein kinase
ANIT	Alpha-naphthylisothiocyanate
ApoB	Apolipoprotein B
ARE	Antioxidant response elements
α-SMA	Alpha-smooth muscle actin
ATF4	Activating Transcription Factor 4
BDL	Bile duct ligation
BTB	Broad complex, tramtrack and bric-a-brac
β-TrCP	Beta-transducin repeat-containing protein
C/EBPα	CCAAT/enhancer-binding protein alpha
CAT	Catalase
CBP	cAMP response element-binding protein (CREB)-binding protein
CCl_4_	Carbon tetrachloride
CHD6	Chromo-ATPase/helicase DNA-binding protein 6
CPT-1A	Carnitine palmitoyltransferase-1A
CREB	cAMP response element-binding protein
CUL	Cullin
CYP2E1	Cytochrome P450 2E1
DGR	Double-glycine repeats
DNMT1	DNA methyltransferase 1
ECM	Extracellular matrix
ER	Endoplasmic reticulum
EZH2	Histone-lysine *N*-methyltransferase enhancer of zeste homolog 2
FABP4	Fatty acid-binding protein 4
FAS	fatty acid synthase
FFAs	Free fatty acids
FGF21	Fibroblast growth factor 21
FXR	Farnesoid X receptor
G6PD	Glucose-6-phosphate dehydrogenase
GCLC	Glutamate-cysteine ligase catalytic
GCS	Gamma-glutamylcysteine synthetase
GPx	Glutathione peroxidase
GR	Glutathione reductase
GSK3	Glycogen synthase kinase 3
GST	Glutathione-S-transferase
HBsAg	Hepatitis B surface antigen
HBV	Hepatitis B virus
HBx	Proteins of HBV
HCB	Hepatitis C virus
HCC	Hepatocellular carcinoma
HDAC	Histone deacetylase
HFD	High-fat diet
HO-1	Hemeoxygenase-1
HSCs	Hepatic stellate cells
HSF1	Heat shock factor 1
Hsp70	Heat shock protein 70
IHBs	Intracellular hyaline bodies
IVR	Intervening region
JNK	c-Jun N-terminal kinase
K8	Keratin 8
KCs	Kupffer cells
Keap1	Kelch-like ECH-associated protein 1
LCA	Lithocholic acid
LXRα	Liver X receptor-alpha
Maf	Musculoaponeurotic fibrosarcoma oncogene homolog
MAFLD	Metabolic dysfunction-associated fatty liver disease
MCD	Methionine- and choline-deficient
MDBs	Mallory-Denk bodies
MMP9	Matrix metalloproteinase 9
MRP	Multidrug resistance associated protein
MTP	Microsomal triglyceride transfer protein
MTTP	Microsomal triglyceride transfer protein
NAC	N-acetyl cysteine
NASH	Non-alcoholic steatohepatitsis
Neh	Nrf2-ECH homology
NF-κB	nuclear transcription factor kappa B
NQO1	NAD(P)H quinone oxidoreductase 1
Nrf2	Nuclear factor erythroid 2-related factor
OPZ	Thiol-reactive agent oltipraz
PDG	Phosphogluconate dehydrogenase
PFD	Pirfenidone
PGC-1α	Peroxisome proliferator-activated receptor gamma coactivator-1 alpha
PPARα	Peroxisome proliferator activated receptor alpha
PPARγ	Peroxisome proliferator activated receptor gamma
RAC3	receptor-associated coactivator 3
Rbx	Ring box protein
Roc1	Homeobox-leucine zipper protein
ROS	Reactive oxygen species
RSV	Resveratrol
SCD1	Stearoyl coenzyme-A desaturase 1
SetD7	SET domain containing 7 histone-lysine *N*-methyltransferase
SIRT1	Sirtuin 1
Skp1	S-phase kinase-associated protein 1
SOD	Superoxide dismutase
SQSTM1	Sequestosome 1
SREBP-1c	Sterol regulatory element-binding protein-1c
TBE31	Acetylenic tricyclic bis (cyano enone)
TGF-β1	Transforming growth factor beta 1
TNFα	Tumoral necrosis factor alpha
Trx	Thioredoxins
TXN1	Thioredoxin 1
UbcM2	E2-ubiquitin conjugating enzyme
UDCA	Ursodeoxycholic acid
ULK1	Unc-51 Like autophagy activating kinase 1
UPR	Unfolded protein responses
VLDL	Very low-density lipoprotein
